# Small-fibre neuropathy in coeliac disease: a cross-sectional study

**DOI:** 10.3389/fneur.2026.1858125

**Published:** 2026-08-14

**Authors:** Livia Sophie Lang, Panoraia Baka, Nikola Hauke, Hüseyin Uysal, Majd Abusaada, Marianne Hahn, Cora Rebhorn, Clemens Sommer, Frank Birklein, Violeta Dimova

**Affiliations:** 1 Department of Neurology, University Medical Center of the Johannes Gutenberg-University Mainz, Mainz, Germany; 2 Institute of Neuropathology, University Medical Center of the Johannes Gutenberg-University Mainz, Mainz, Germany

**Keywords:** coeliac disease, intraepidermal nerve fibre density, neuropathic pain, quantitative sensory testing, skin biopsy, small fibre neuropathy

## Abstract

**Introduction:**

Coeliac disease (CD) is associated with neurological manifestations, including peripheral neuropathy. While sensorimotor neuropathy has been described, objective evidence for small-fibre neuropathy (SFN) in CD remains limited. We aimed to assess large- and small-fibre involvement in patients with CD with and without neuropathic symptoms using contemporary diagnostic standards.

**Methods:**

In this cross-sectional study, 36 adults with biopsy-proven CD were recruited nationwide and stratified by the Neuropathy Symptom Score into symptomatic (pwCD(NS+), NSS ≥ 3) and asymptomatic (pwCD(NS−), NSS < 3) groups. All participants underwent neurological examination, sural nerve conduction studies (NCS), quantitative sensory testing (QST), distal leg skin biopsy for intraepidermal nerve fibre density (IENFD), and autonomic testing.

**Results:**

pwCD(NS+) predominantly reported distal symmetric sensory complaints with neuropathic pain characteristics. NCS were normal in both groups, arguing against relevant sensorimotor neuropathy. Both groups showed a loss-of-function QST profile with impaired thermal detection. IENFD was lower in pwCD(NS+) than in pwCD(NS−) and correlated with symptom severity. Autonomic testing was largely unremarkable. 50% of pwCD(NS+) fulfilled the diagnostic criteria for SFN, while all remaining patients showed abnormalities in at least one small fibre-related domain (clinical examination, IENFD or QST). In pwCD(NS−), isolated abnormalities in QST or IENFD were frequently observed despite the absence of neuropathic symptoms.

**Conclusion:**

In CD, neuropathic symptoms are associated with small-fibre pathology rather than sensorimotor neuropathy. SFN should be considered in the diagnostic work-up of neuropathic complaints in CD, and symptom-oriented treatment is warranted.

## Introduction

Coeliac disease (CD) is a chronic immune-mediated enteropathy with a global prevalence of approximately 1% (1, 2). In genetically predisposed individuals, gluten ingestion induces an aberrant immune response involving gluten-specific CD4^+^ T cells and disease-specific autoantibodies, leading to intestinal inflammation and villous atrophy (3). Clinically, this results in gastrointestinal manifestations such as diarrhea, weight loss, and malnutrition (4).

Beyond the gastrointestinal tract, CD is associated with a broad spectrum of extraintestinal manifestations. Some of these are attributable to malabsorption-related deficiencies, including osteoporosis and anemia (5, 6). However, other manifestations cannot be explained by nutrient deficiency alone and are thought to reflect systemic immune activation (4–7), potentially facilitated by increased intestinal permeability and disruption of the gut-blood barrier (3, 8–10). Immune-mediated pathology has been demonstrated in several organs, including the skin and the nervous system, exemplified by dermatitis herpetiformis and neurological involvement affecting the central and peripheral nervous system (7, 11–15).

These neurological symptoms were first recognized with the description of gluten ataxia in 1966 (16). Later, axonal sensorimotor peripheral neuropathy (PNP) was described as another clinical presentation (17, 18). However, most studies on peripheral neuropathic symptoms have investigated the prevalence of CD in patients already diagnosed with PNP, often without using standardized diagnostic procedures and with inconsistent consideration of vitamin deficiencies as confounders (14, 19–22). Consequently, the strength and frequency of this association remain debated, and population-based data suggest that sensorimotor neuropathy in CD is relatively uncommon (23, 24). Nevertheless, current National Institute for Health and Care Excellence guidelines recommend serological testing for CD in patients presenting with PNP (25).

Clinically, patients with CD often report neuropathic pain and paraesthesia (26). While such symptoms may occur in PNP, they are also characteristic of small-fibre neuropathy (SFN), which selectively affects thinly myelinated Aδ- and unmyelinated C-fibres and is not captured by routine nerve conduction studies. SFN is also a common feature of other autoimmune disorders such as systemic lupus erythematosus or Sjögren’s syndrome (27, 28). However, direct evidence for SFN in CD is limited to a single case series demonstrating reduced intraepidermal nerve fibre density in eight patients (29).

## Materials and methods

### Study design

In view of this, we conducted a cross-sectional study at the Department of Neurology, University Medical Center of the Johannes Gutenberg University Mainz, Germany to establish whether patients with coeliac disease (pwCD) with and without neuropathic symptoms show objective evidence of neuropathy, and whether large- or small-fibre involvement predominates. The study was approved (approval number 837.224.15) and conducted in accordance with the Declaration of Helsinki.

### Coeliac disease cohort

PwCD were recruited in collaboration with the German Coeliac Society (DZG). Based on the Neuropathy Symptom Score (NSS) (30), patients were classified as pwCD with neuropathic symptoms (NSS ≥ 3; pwCD(NS+)) or without/minimal symptoms (NSS < 3; pwCD(NS−)) (31) (for flow chart, see Supplementary Figure S1). Inclusion criteria were: (i) confirmed diagnosis of coeliac disease (CD) according to the German guideline, verified by clinical records and histopathology, (ii) age ≥18 years, and (iii) current adherence to a strict gluten-free diet, minimizing the likelihood of malnutrition-related neuropathy. Exclusion criteria included history of malignancy, acute or chronic infection, autoimmune disorders other than CD, diabetes mellitus, vitamin B12 deficiency, pregnancy, and lactation All participants underwent a standardized neurological examination by a board-certified physician (FB) and structured assessment of neuropathic and autonomic symptoms. Neuropathic pain was evaluated using the painDETECT® and DN4 questionnaires, and psychological symptoms were assessed with the Hospital Anxiety and Depression Scale (HADS). Peripheral nerve function was evaluated using nerve conduction studies (NCS), quantitative sensory testing (QST), skin biopsy for quantification of intraepidermal nerve fibre density (IENFD), and autonomic function testing.

### Nerve conduction studies (NCS)

All patients underwent sensory NCS of the sural nerve, which served as a sensitive marker of large fibre involvement (32, 33). Antidromic recordings were performed in a temperature-controlled room using standard techniques (34). Results were compared with our laboratory’s reference values (sensory nerve action potential (SNAP) amplitude ≥5 μV; nerve conduction velocity (NCV) ≥ 40 m/s). In line with EFNS/PNS criteria (35) axonal changes were defined as a reduction of the SNAP amplitude (baseline to first negative peak) below the lower limit of normal.

### Quantitative sensory testing (QST)

QST was performed unilaterally at the right hand and on the right foot using TSA-II device, (Medoc Ltd., Israel, Somedic SenseLab AB) according to previously published protocol of the German research network of neuropathic pain (DFNS) (36). The test battery included thermal detection and pain thresholds, and paradoxical heat sensations (PHS) with alternating stimuli. Raw data were transformed into z-scores based on published age- and sex-adjusted reference values as previously described (37). QST was considered indicative for SFN when cold detection threshold (CDT) or warm detection threshold (WDT) at the foot were outside the reference range, as these parameters are most sensitive for SFN (36, 38).

### Skin biopsy

Skin punch biopsies were taken 10 cm proximal to the right lateral malleolus under local anesthesia using a disposable 3-mm punch. Samples were processed for quantification of intraepidermal nerve fibre density (IENFD) following established international standards (39, 40). Fibre counts were performed according to validated counting rules (40) by an investigator blinded to group allocation, and results were independently reviewed by an experienced neurologist (PB), also blinded to clinical status.

### Autonomic function testing

Autonomic neuropathy was assessed by testing heart-rate variability. Four statistical values in the time domain were obtained at rest and during the Valsalva maneuver (41).

### Statistical analysis

Statistical analyses were performed using IBM SPSS 23 Statistics, version 23.0, GraphPad Prism 9 and R version 4.4.2 for Windows. QST parameters were log-transformed where appropriate and z-standardized using published DFNS reference values stratified by age, sex, and body region (36). For QST, group-level z-values were compared against the normative reference value of 0 using two-sided one-sample Wilcoxon signed-rank tests. Between-group comparisons of QST parameters were performed using two-sided Wilcoxon rank-sum tests. Bonferroni correction was applied for multiple comparisons where indicated. Between-group comparisons of other continuous variables (e.g., vibration thresholds, HRV parameters, IENFD, HADS scores) were performed using two-sided Wilcoxon rank-sum tests. Categorical variables, including the presence of paradoxical heat sensations (PHS) and the proportion of patients with abnormal findings in each diagnostic domain (clinical signs, foot QST, and IENFD), were analysed using Fisher’s exact test. Data distribution was assessed by visual inspection and the D’Agostino-Pearson normality test. Continuous data are reported as mean ± SD unless otherwise stated. Associations between clinical symptom severity (Neuropathy Symptom Score, NSS) and psychological, sensory, or morphological measures were assessed using two-sided Spearman’s rank correlation coefficients. Given the exploratory nature of these analyses, no adjustment for multiple comparisons was applied. A two-sided *p*-value < 0.05 was considered statistically significant unless otherwise specified for multiple testing. For individual-level interpretation, values were considered abnormal if they fell outside the 95% confidence interval of age-adjusted normative reference data (37).

## Results

### Study population and demographics

According to the Neuropathy Symptom Score (NSS), 21 patients with coeliac disease were classified as having neuropathic symptoms (pwCD(NS+)) and 15 as without/minimal symptoms (pwCD(NS−)). No significant differences in age or sex distribution were observed between the two groups. In both pwCD groups, classical CD (characterized by abdominal distension, gastrointestinal complaints, and malnutrition) was the most common presentation, followed by non-classical disease (predominantly extraintestinal manifestations such as chronic hepatitis or mood changes) and rarely silent disease (positive serology and duodenal biopsy in the absence of symptoms); (see Table 1 for details). At the time of study participation, all pwCD were on a strict gluten-free diet and self-reported clinical remission.

**Table 1:** Demographic and clinical characteristics of patients with coeliac disease (pwCD), stratified by neuropathic symptoms according to the Neuropathy Symptom Score (NSS).

Variable	pwCD (NS+) (*N* = 21)	pwCD (NS−) (*N* = 15)
Age (mean ± SD)	49.2 ± 11.5 years	46.1 ± 11.7 years
Gender (female)	66.7% (*N* = 14)	55.3% (*N* = 8)
Classical CD	52.4% (*N* = 11)	55.3% (*N* = 8)
Silent CD	4.8% (*N* = 1)	13.3% (*N* = 2)
Non-Classical CD	38.0% (*N* = 8)	33.3% (*N* = 5)
Duration of CD (mean ± SD)	140.6 ± 132 months	124.9 ± 71.7 months
Age at Diagnosis of CD (mean ± SD)	37 ± 14.1 years	35.7 ± 10 years

Data are presented as mean ± SD or percentage (%). CD, coeliac disease; NSS, Neuropathy Symptom Score; pwCD, patients with coeliac disease; NS, neuropathic symptoms; SD, standard deviation.

### Reported neuropathic symptoms in pwCD

**Figure 1:**
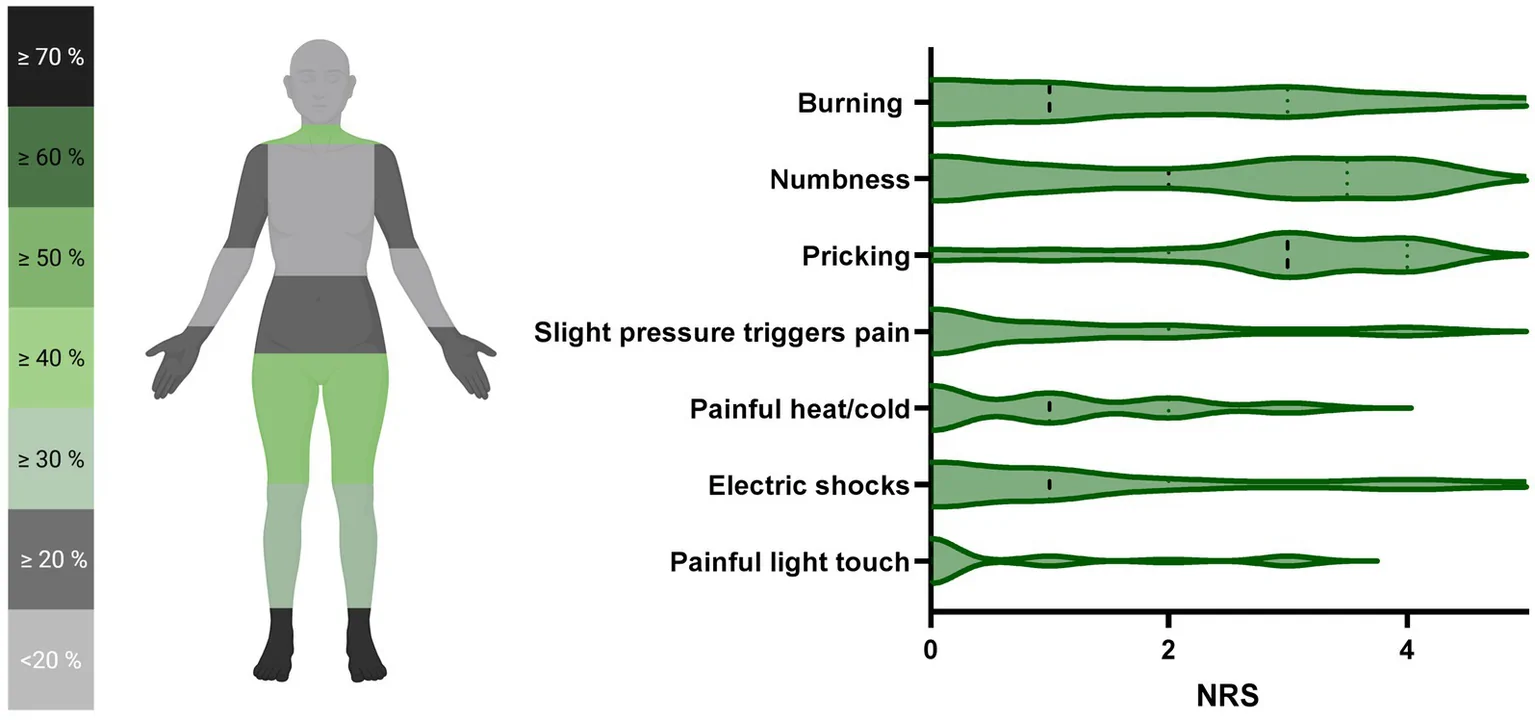
Neuropathic symptom profiles in patients with coeliac disease and neuropathic symptoms assessed by painDETECT®. Left: Body map illustrating the percentage of patients reporting symptoms in each body region. Right: Intensity of neuropathic symptom descriptors shown as violin plots of numerical rating scale (NRS) scores. NRS = numerical rating scale. Created with BioRender.com.

In pwCD(NS+), the structured interview most frequently revealed paraesthesia (71.4%), pain (61.9%), and numbness (47.6%). Weakness (33.3%) and cramps (28.5%) were also reported. Autonomic complaints were present in 33% of patients, including constipation (23.8%), syncope (9.5%), and urinary incontinence (14.2%); no cases of hypo−/anhidrosis or erectile dysfunction were reported. Analgesic treatment was uncommon: no patients received NSAIDs or opioids, and only 9.5% reported use of anti-neuropathics. In pwCD(NS−), no sensory symptoms were reported and autonomic complaints were less frequent (Table 2 for details). In pwCD(NS+), mean pain intensity at rest was 2.5 ± 2.5 on a numerical rating scale (NRS, 0–10), compared with 0 in pwCD(NS−). All pwCD(NS+) with pain fulfilled criteria for neuropathic pain according to the DN4. Symptom characteristics were further assessed with the painDETECT® questionnaire. The body map analysis showed a mainly distal-symmetric distribution, with the lower limbs (thighs, knees, calves, and feet) most frequently affected, followed by the upper limbs and, less commonly, the trunk. Regarding sensory descriptors the highest ratings and the most frequently reported symptoms were burning, tingling, and numbness, followed by electric-shock-like sensations, painful pressure, and thermal discomfort (Figure 1).

**Table 2:** Reported symptoms of patients with coeliac disease (pwCD) with (NS+) and without (NS−) neuropathic symptoms, assessed by structured interview.

Variable	pwCD (NS+) (*N* = 21)	pwCD (NS−) (*N* = 15)
Pain (yes)	61.9% (*N* = 13)	0.0% (*N* = 0)
Burning (yes)	52.3% (*N* = 11)	0.0% (*N* = 0)
Numbness (yes)	47.6% (*N* = 10)	0.0% (*N* = 0)
Paresthesia (yes)	71.4% (*N* = 15)	0.0% (*N* = 0)
Weakness (yes)	33.3% (*N* = 7)	0.0% (*N* = 0)
Cramps (yes)	28.5% (*N* = 6)	0.0% (*N* = 0)
Constipation (yes)	23.8% (*N* = 5)	33.3% (*N* = 5)
Syncope (yes)	9.5% (*N* = 2)	6.6% (*N* = 1)
Urinary incontinence (yes)	14.2% (*N* = 3)	0.0% (*N* = 0)
Hypo/Anhidrosis (yes)	0.0% (*N* = 0)	0.0% (*N* = 0)
Erectile dysfunction (yes)	0.0% (*N* = 0)	0.0% (*N* = 0)
Duration of NS (mean ± SD)	52.3 ± 45.6 years (25% before CD diagnosis)	N/A
NSAID (yes)	0.0% (*N* = 0)	0.0% (*N* = 0)
Opioids (yes)	0.0% (*N* = 0)	0.0% (*N* = 0)
Neuropathic pain agents (yes)	9.5% (*N* = 2)	0.0% (*N* = 0)

Data are presented as percentage (%) and absolute numbers (N). Duration of neuropathic symptoms is given as mean ± SD. CD, coeliac disease; pwCD, patients with coeliac disease; NS, neuropathic symptoms; *N*, number of patients; NSAID, non-steroidal anti-inflammatory drug; N/A, not applicable; SD, standard deviation.

### Assessment of large fibre function

**Figure 2:**
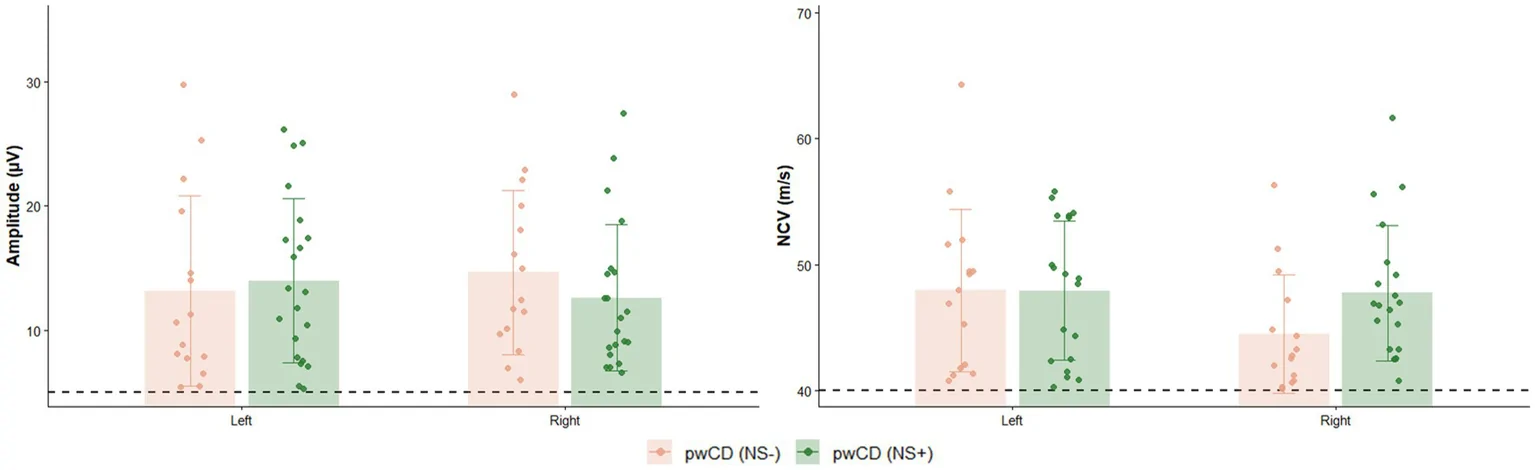
Sural sensory nerve action potential (SNAP) amplitudes (left) and conduction velocities (NCV; right) in patients with coeliac disease and neuropathic symptoms (pwCD(NS+)) and those without (pwCD(NS−)). The dotted lines indicate our EMG laboratory reference values (amplitude ≥5 μV, velocity ≥40 m/s).

Clinical neurological and electrophysiological examination was performed to assess signs of large fibre neuropathy, including muscle strength, tendon reflexes, position sense, vibration perception, and sural NCS bilaterally (see Table 3 for the most sensitive tests, and Supplementary Table S1 for the complete examination protocol). In both patient groups, abnormalities were rare. Vibration thresholds did not differ significantly between groups (Wilcoxon rank-sum, all *p* > 0.05). In NCS (Figure 2), both sural SNAP and NCV were within the normal range in both groups, with no significant between-group differences for sural SNAP amplitudes on either side (all *p*_adj > 0.05) or for NCV on the left side (*p*_adj = 1.00).

**Table 3:** Neurophysiological and clinical findings in patients with coeliac disease with neuropathic symptoms (pwCD(NS+)) and without neuropathic symptoms (pwCD(NS−)).

Variable	pwCD (NS+) (*N* = 21)	pwCD (NS−) (*N* = 15)
Pathological Sural SNAP	0.0% (*N* = 0)	0.0% (*N* = 0)
Pathological Sural NCV	0.0% (*N* = 0)	0.0% (*N* = 0)
Reduced Achilles tendon reflexes	9.5% (*N* = 2)	0.0% (*N* = 0)
Foot Dorsiflexion Paresis	0.0% (*N* = 0)	0.0% (*N* = 0)
Muscle atrophy	0.0% (*N* = 0)	0.0% (*N* = 0)
Vibration threshold—feet	5.6 ± 1.8	5.5 ± 2.5
Vibration threshold—hand	6.4 ± 1.1	6.9 ± 1.0
Impaired position sense—toe	4.8% (*N* = 1)	0.0% (*N* = 0)

Data are presented as mean ± SD for continuous variables and percentage (%) of affected patients for categorical variables. Vibration perception threshold is reported on a 0–8 scale (arbitrary units). CD, coeliac disease; pwCD, patients with coeliac disease; NS, neuropathic symptoms; SNAP, sensory nerve action potential; NCV, nerve conduction velocity; SD, standard deviation.

### Assessment of small fibre function

**Figure 3:**
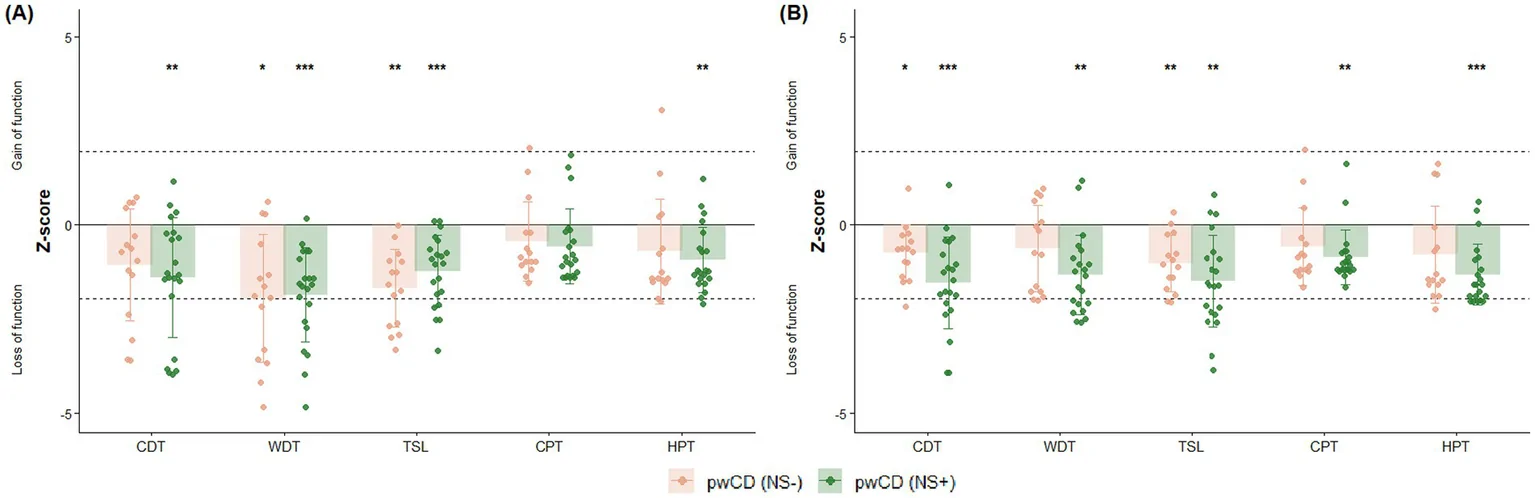
Quantitative sensory testing (QST) results in patients with coeliac disease with neuropathic symptoms (pwCD(NS+)) and without neuropathic symptoms (pwCD(NS−)). Data are presented as *z*-scores (mean ± *SD*) for cold detection threshold (CDT), warm detection threshold (WDT), thermal sensory limen (TSL), cold pain threshold (CPT), and heat pain threshold (HPT). Panel **(A)** shows the control site (hand), and panel **(B)** the test site (foot). Negative *z*-scores indicate loss of function, whereas positive *z*-scores indicate gain of function. Dashed lines represent the 95% confidence interval of the normative reference (±1.96). Significance levels indicate deviations from zero within each group assessed using the Wilcoxon signed-rank test and were corrected for multiple comparisons using the Bonferroni method applied separately within each group and anatomical site. *p < 0.05, **p < 0.01; ***p < 0.001.

Quantitative sensory testing (QST) demonstrated a predominantly loss-of-function profile in both pwCD(NS+) and pwCD(NS−), affecting thermal detection thresholds and selected pain measures (Figure 3).

Compared to the normative reference (*z* = 0), pwCD(NS+) showed significant reductions across multiple parameters at both sites, whereas pwCD(NS−) exhibited fewer deviations from zero. At the hand (control site), pwCD(NS+) demonstrated significant loss of function for CDT (*p*_adj = 0.006), WDT (*p*_adj < 0.001), TSL (*p*_adj < 0.001), and HPT (*p*_adj = 0.004) (Wilcoxon signed-rank test, Bonferroni-corrected), while pwCD(NS−) showed significant deviations only for WDT (*p*_adj = 0.014) and TSL (*p*_adj = 0.004) (Wilcoxon signed-rank test, Bonferroni-corrected).

At the foot (test site), pwCD(NS+) exhibited significant loss of function across all thermal detection parameters as well as pain thresholds (all *p*_adj ≤ 0.006) (Wilcoxon signed-rank test, Bonferroni-corrected), whereas pwCD(NS−) showed significant deviations from zero only for CDT (*p*_adj = 0.025) and TSL (*p*_adj = 0.010) (Wilcoxon signed-rank test, Bonferroni-corrected). Overall, this pattern indicates more pronounced sensory alterations in pwCD(NS+) compared to pwCD(NS−), although direct between-group comparisons did not reach statistical significance after correction for multiple testing (Wilcoxon rank-sum test, all *p*_adj > 0.3).

Paradoxical heat sensations (PHS) were more frequent in pwCD(NS+) (12/21; 57.1%) than in pwCD(NS−) (4/15; 26.7%), but this difference did not reach statistical significance (Fisher’s exact test, two-sided *p* = 0.096). Upper-limb PHS occurred in 4/21 (19.0%) and 3/15 (20.0%), respectively (Fisher’s exact test, *p* > 0.99).

Heart rate variability (HRV) parameters were within the normal range across groups. No significant between-group differences were observed for any autonomic parameter (Wilcoxon rank-sum test; all *p* > 0.05).

**Figure 4:**
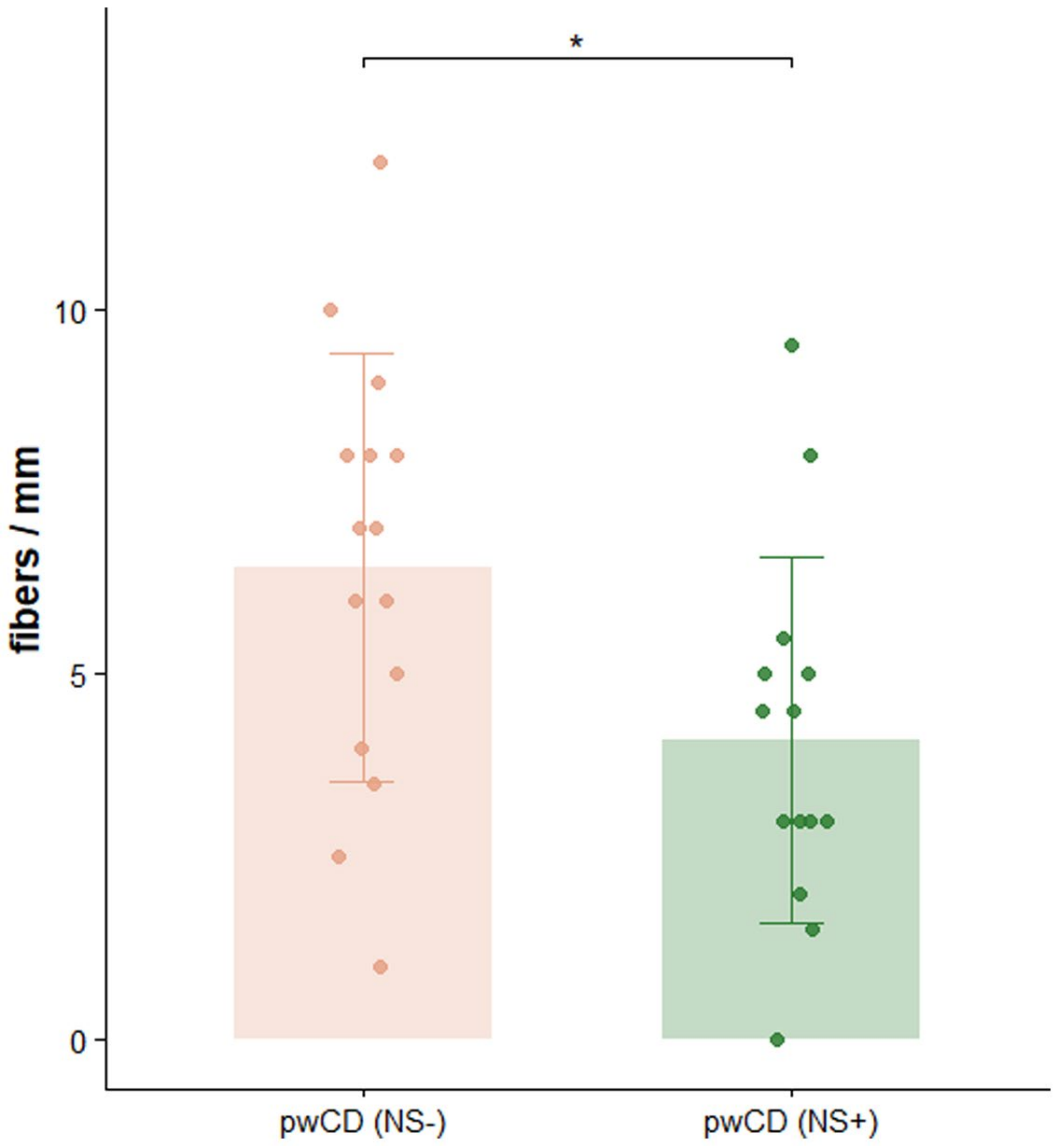
Intraepidermal nerve fibre density (IENFD) in patients with coeliac disease and neuropathic symptoms (pwCD(NS+)), patients with coeliac disease without neuropathic symptoms (pwCD(NS−)). Data are shown as individual values with mean ± SD. Wilcoxon rank-sum test *p < 0.05.

Intraepidermal nerve fibre density (IENFD) was significantly lower in pwCD (NS+) than in pwCD (NS−) (Wilcoxon rank-sum test, *p* = 0.032; Figure 4).

### SFN classification

**Figure 5:**
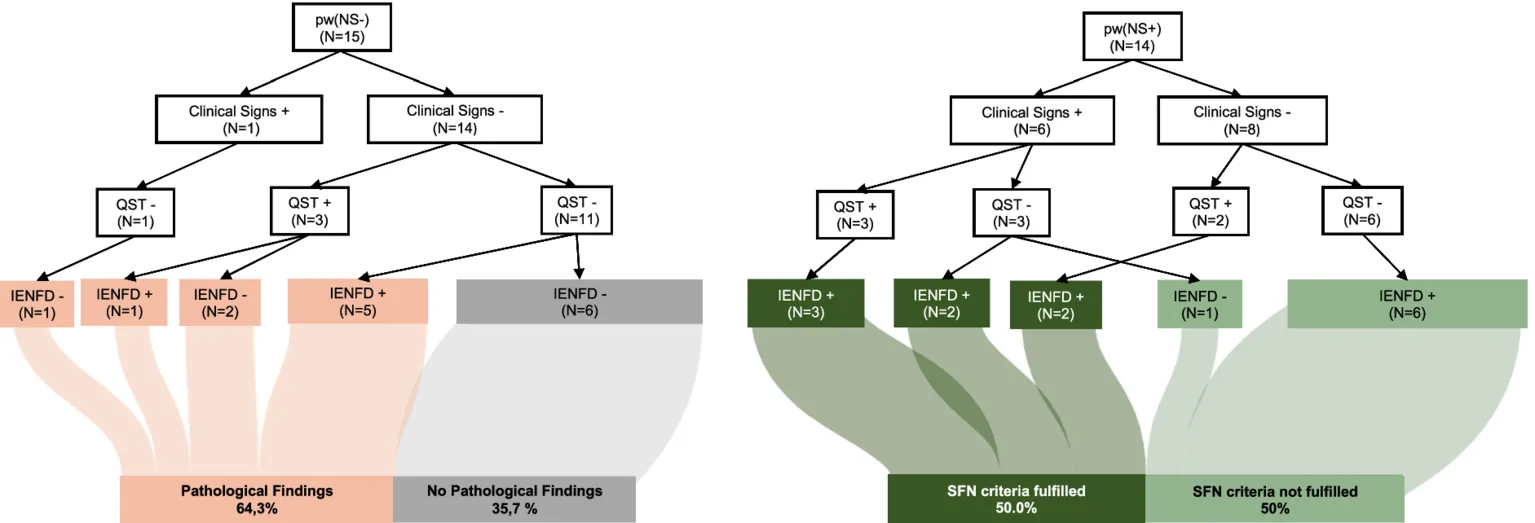
Diagnostic workflow for small fibre neuropathy (SFN) assessment in patients with coeliac disease with (pwCD(NS+)) and without neuropathic symptoms (pwCD(NS−)). The workflow integrates clinical examination, quantitative sensory testing (QST), and intraepidermal nerve fibre density (IENFD). SFN classification according to the Devigili/Besta criteria was performed only in pwCD(NS+) with complete datasets (*N* = 14). In pwCD(NS−), the diagram illustrates the distribution of abnormal findings, which were not considered diagnostic of SFN in the absence of neuropathic symptoms and a compatible clinical phenotype. “+” denotes an abnormal finding and “-” a normal finding within the respective diagnostic domain. Created with SankeyMATIC.com.

Classification followed the widely accepted Besta criteria proposed by Devigili et al. (38). Accordingly, pwCD(NS+) with a compatible clinical history and normal NCS were classified as having SFN if at least two of the following three domains were abnormal: (i) clinical signs of small-fibre dysfunction (pinprick and/or thermal hypoesthesia, allodynia, and/or hyperalgesia); (ii) abnormal QST (CDT and/or WDT) at the feet; and (iii) reduced IENFD at the distal leg according to the normative values reported by Lauria et al. (40). Detailed numbers of abnormal findings for each diagnostic domain are provided in Supplementary Table S2; the corresponding diagnostic workflow is shown in Figure 5.

Using this classification, 7/14 (50.0%) pwCD(NS+) with complete datasets fulfilled the diagnostic criteria for SFN. None of the remaining patients had entirely unremarkable findings; all showed abnormalities in at least one diagnostic domain but did not meet the threshold of two abnormal domains required for SFN classification. Seven pwCD(NS+) did not undergo skin biopsy and therefore lacked IENFD data. Nevertheless, 4/7 (57.1%) fulfilled both remaining diagnostic domains (clinical examination and QST) and were therefore classified as having SFN, whereas the remaining 3/7 (42.9%) could not be classified unequivocally owing to the missing IENFD data. In pwCD(NS−) 9/15 (64.3%) exhibited at least one abnormal small-fibre domaine. However, in the absence of neuropathic symptoms or a compatible clinical phenotype, these isolated abnormalities were not considered diagnostic for SFN. Between-group comparison of individual small-fibre test abnormalities revealed that pwCD(NS+) showed significantly higher rates of pathological clinical signs (9/21, 42.8% vs. 1/15, 6.7%; Fisher’s exact test, OR = 9.91, *p* = 0.024) and reduced IENFD (13/14, 92.8% vs. 6/15, 40.0%; Fisher’s exact test, OR = 17.40, *p* = 0.005) compared to pwCD(NS−). The proportion of patients with pathological foot QST (CDT or WDT) did not differ significantly between groups (9/21, 42.8% vs. 3/15, 20.0%; Fisher’s exact test, OR = 2.91, *p* = 0.282).

### Psychological aspects

Anxiety and depressive symptoms were common in both patient groups. Continuous HADS scores did not differ significantly between pwCD(NS+) and pwCD(NS−), neither for anxiety (HADS-A: Wilcoxon rank-sum test, *p* = 0.579) nor for depressive symptoms (HADS-D: *p* = 0.358) (Supplementary Figure S2).

Using the clinical cut-off (≥11) (42), elevated anxiety symptoms were present in 12/15 pwCD(NS−) (80.0%) and 20/21 pwCD(NS+) (95.2%). Elevated depressive symptoms were less frequent, observed in 1/15 pwCD(NS−) (6.7%) and 7/21 pwCD(NS+) (33.3%).

### Associations between symptom severity and clinical measures

To assess the relationship between clinically reported neuropathic complaints and other variables, we correlated the Neuropathy Symptom Score (NSS) with psychological, sensory, and neurobiological measures using Spearman’s rank correlation. As expected NSS was positively associated with pain intensity (NRS; *ρ* = 0.701, *p* < 0.001). Higher NSS was further associated with lower intraepidermal nerve fibre density (IENFD; *ρ* = −0.441, *p* = 0.017). NSS also showed a borderline significant inverse association with cold detection threshold (CDT; *ρ* = −0.323, *p* = 0.055). No significant associations were observed between NSS and HADS-D, HADS-A, remaining QST, or nerve conduction studies (all *p* > 0.05). To assess the potential influence of psychological distress on diagnosis-relevant small-fibre measures, HADS-A and HADS-D were correlated with CDT, WDT, and IENFD using Spearman’s rank correlation. No associations were observed (HADS-A: *ρ* = 0.165, *p* = 0.344; ρ = 0.087, *p* = 0.620; *ρ* = −0.030, *p* = 0.879; HADS-D: *ρ* = −0.234, *p* = 0.176; *ρ* = −0.283, *p* = 0.099; *ρ* = 0.044, *p* = 0.825, for CDT, WDT, and IENFD respectively), arguing against a relevant confounding effect of psychological distress on the observed sensory and morphological findings.

## Discussion

Our study provides the first systematic characterization of neuropathy in a German wide recruited cohort of pwCD using contemporary diagnostic standards. By combining clinical, neurophysiological and morphological assessments, we clarified that a significant subgroup of pwCD suffers from SFN while sensorimotor neuropathy was the exception.

### Clinical presentation

The complaints of pwCD(NS+) were mainly sensory disturbances. Pain was most frequently described as burning and tingling in a distal, symmetrical distribution predominantly affecting the feet. Pain intensity was modest. According to the results presented herein, the origin of the pain must now be classified as neuropathic and should be treated accordingly. Currently, this is only the case in a minority of pwCD(NS+). In some patients, pain extended beyond the typical distal distribution. Together with the frequent upper-limb QST abnormalities, a non-length-dependent pattern cannot be excluded (43). Alternatively, pain might be independent from neuropathy in these cases. Numbness was also a common symptom, which could not always be confirmed by neurological investigation, and vibration threshold was close to normal. Weakness was reported in about one-third of patients but none of the patients had foot paresis or muscular atrophy. Interestingly, one-third of pwCD reported autonomic complaints (e.g., constipation or syncope); however, no pathological values were detected on heart rate variability or cardiovascular reflex tests. In general, combination of symptoms and findings in clinical examination closely resembled that seen in other coeliac disease—related neuropathy reports (20, 26) or other immune-mediated neuropathies associated with systemic lupus erythematosus or Sjögren’s syndrome (44, 45). Importantly, none of our patients fulfilled the criteria for fibromyalgia, as generalized pain was absent (46).

### Sensorimotor neuropathy

Our results clearly demonstrate that pwCD lack sensorimotor neuropathy. Clinical investigation revealed reduced Achilles tendon reflexes only in two pwCD while motor strength and testing sensory function was unremarkable. In addition, nerve conduction studies were normal in all cases. This contrasts with earlier reports suggesting an increased risk of sensorimotor polyneuropathy in CD. However, population-based data indicate that the absolute prevalence of sensorimotor PNP in CD in general is modest (~0.7% vs. 0.3% in controls) (17, 22). Moreover, most prior studies recruited patients with already diagnosed sensorimotor PNP and subsequently screened for CD (13, 19, 20), thereby enriching their cohort with more affected cases. Other reports describe findings that are difficult to attribute to CD as the leading cause for neuropathy: e.g., abnormalities confined to arm nerves, old patient age, competing PNP causes (47). Our results of negative findings for a sensorimotor PNP phenotype in pwCD seem valid: The cohort was systematically recruited after screening via a national patient organization, and it was examined with standardized diagnostic protocols routinely applied in clinical practice. This means that the sensory and motor complaints of our patients seem to be functional rather than related to peripheral large fibre damage. In previous investigations, we (48) and others (49) have been able to show that numbness and motor weakness can be caused as a functional consequence of pain rather than nerve damage.

### Small fibre involvement

In contrast to sensorimotor neuropathy, we found convergent evidence for small-fibre pathology in a substantial proportion of pwCD with neuropathic symptoms. According to established Devigili/Besta criteria (38), 50% of pwCD(NS+) fulfilled the criteria for SFN while the remainder showed abnormalities in at least one diagnostic domain. QST demonstrated reduced thermal detection, particularly for cold stimuli at both the feet and hands, suggesting small-fibre dysfunction of Aδ- and C-fibres (36, 50), which is further confirmed by reduced IENFD. Interestingly, IENFD correlated with a higher prevalence of neuropathic symptoms (high NSS), which underlines the clinical relevance of small fibre loss for neuropathic complains. The autonomic fibres seem not affected in pwCD. More than half of pwCD(NS−) showed abnormalities in QST and/or IENFD. Overall, the degree of dysfunction was less pronounced than in pwCD(NS+). Nevertheless, pwCD(NS−) cannot be classified as having symptomatic SFN in the absence of neuropathic complaints (38). Outside of this study setting, such abnormalities would unlikely to be detected, and their clinical relevance remains uncertain. However, these findings may indicate either limited concordance between individual small fibre measures and SFN symptoms (45) or that pwCD(NS−) and pwCD(NS+) not yet fulfilling the diagnostic criteria for SFN, might represent early stages. While the correlation between IENFD and the NSS is compatible with an early-stage hypothesis, several observations argue against a tight coupling between pathology and symptoms. First, our recruitment strategy did not enrich for neuropathic symptomatology. Second, the known interindividual variability of QST (37) and IENFD (51) may in part reflect physiological variation. Taken together, these data imply that additional factors might determine whether small-fibre lesions translate into pain or remain asymptomatic (52). The mechanisms underlying small-fibre pathology in CD remain unresolved. Potential explanations include transient nutritional deficiencies during periods of untreated disease and/or direct immune-mediated injury. A humoral autoimmune contribution is plausible, as SFN occurs in immune-mediated disorders (e.g., Sjögren’s syndrome, sarcoidosis) and, in idiopathic SFN, candidate autoantibodies have been described (53) although their pathogenic relevance remains debated (54). Experimental work further supports a potential humoral contribution, as passive transfer of IgG from patients with SFN can recapitulate neuropathic symptom-like behavior in mice (55). However, whether such antibody-mediated mechanisms contribute to small-fibre pathology in CD remains unknown. As we did not assess serological or tissue markers of neuronal autoimmunity, our study cannot address this question.

### Affective symptoms and implications for patient care

Our results eventually carry implications for patient care. Both CD groups exhibited elevated levels of psychological distress, with anxiety symptoms predominating, consistent with previous reports in CD (56). However, mean HADS scores were higher than those reported, likely reflecting recruitment through a national patient advocacy organization and the associated enrichment for patients with a higher disease burden. Although rates also appeared higher than those reported in population-based registry studies (57), the latter are not comparable because they report clinically diagnosed psychiatric disorders rather than HADS-based psychological distress. Importantly, HADS scores neither differed between pwCD(NS+) and pwCD(NS−) nor correlated with any measure of small-fibre pathology, arguing against a relevant confounding effect on the observed QST and morphological findings. Despite this, none of our patients received targeted treatment for affective symptoms nor neuropathic complaints. This treatment gap likely reflects both under-recognition of neuropathic manifestations in CD and the fact that many patients remain undiagnosed or insufficiently evaluated in routine clinical practice. Treatment guidelines for neuropathic pain mention serotonine-noradrenaline reuptake inhibitors first line (58), drugs which potentially also improve affective symptoms. SNRIs may therefore represent a pragmatic option that addresses both symptom domains.

### Limitations

Despite representing the largest controlled study in literature systematically investigating neuropathy in CD, the sample size remains modest. Furthermore, selection bias cannot be excluded as recruitment was based on patient-organization announcements. We also did not assess serological or tissue markers of anti-neuronal autoimmunity, which limits mechanistic insights. Skin biopsies were restricted to distal sites; proximal biopsies would have been required to definitely distinguish between length-dependent and non-length-dependent SFN. Nerve conduction studies were limited to the sural nerve and thus to the lower extremity. However, given that the predominant clinical phenotype was sensory and length-dependent, it appears unlikely that relevant abnormalities would have been confined to motor nerves or exclusively to the upper extremities in the presence of normal sural recordings. Finally, the high prevalence of anxiety and depressive symptoms in both CD groups may have influenced symptom reporting and QST results, potentially contributing to lower detection thresholds.

## Conclusion

PwCD(NS+) frequently meet criteria for SFN. Conversely, CD should be addressed in the diagnostic work-up of patients with SFN of unknown origin in specialized centers. Efforts should focus on investigating autoimmune mechanisms responsible for the pathophysiology of SFN in pwCD because only then a targeted treatment is possible. Until this has been achieved, a symptom-guided treatment of neuropathic symptoms in pwCD is warranted.

## Data Availability

The raw data supporting the conclusions of this article will be made available by the authors, without undue reservation.

## Supplementary material

SUPPLEMENTARY FIGURE S1 Recruitment of patients with coeliac disease (pwCD). In total, 312 individuals responded to the study announcement, 127 returned the questionnaire, and 36 fulfilled all criteria for inclusion. Patients were stratified by the Neuropathy Symptom Score (NSS) into pwCD-NS+ (NSS ≥ 3; *n* = 21) and pwCD-NS− (NSS < 3; *n* = 15).

SUPPLEMENTARY FIGURE S2 Depressive (HADS-D) and anxiety (HADS-A) symptoms across groups. Bars represent group means with standard error of the mean (SEM); individual data points are shown. The dotted line indicates the clinical cut-off (≥11). Groups: pwCD(NS−) = patients with coeliac disease without neuropathic symptoms; pwCD(NS+) = patients with coeliac disease with neuropathic symptoms.

SUPPLEMENTARY TABLE S1 Clinical neurological examination findings (deep tendon reflexes and focal paresis) in patients with coeliac disease with neuropathic symptoms (pwCD(NS+)) and without neuropathic symptoms (pwCD(NS−)). Data are presented as percentage (%) of patients with abnormal findings. CD = coeliac dis-ease; pwCD = patients with coeliac disease; NS = neuropathic symptoms.

SUPPLEMENTARY TABLE S2 Clinical sensory findings and small-fibre assessments in patients with coeliac disease with neuropathic symptoms (pwCD(NS+)) and without neuropathic symptoms (pwCD(NS−)). Data are presented as percentage (%) of patients with patho-logical findings. Pathological QST (CDT, WDT) was defined as z-scores outside ±1.96; pathological IENFD as reduced intraepidermal nerve fibre density; pathological AFT as abnormal autonomic function testing. AFT = autonomic function test-ing; CD = coeliac disease; CDT = cold detection threshold; IENFD = intraepidermal nerve fibre density; NS = neuropathic symptoms; pwCD = patients with coeliac dis-ease; QST = quantitative sensory testing; WDT = warm detection threshold.
