# Supplementary material for: Small-fibre neuropathy in coeliac disease: a cross-sectional study
Source: Front Neurol. 2026 Aug 14;17:1858125. doi: 10.3389/fneur.2026.1858125 (PMC13521803; doi:10.3389/fneur.2026.1858125)
Supplement: Supplementary file 1 [file Data_Sheet_1.pdf]

Suppl. Figure 1

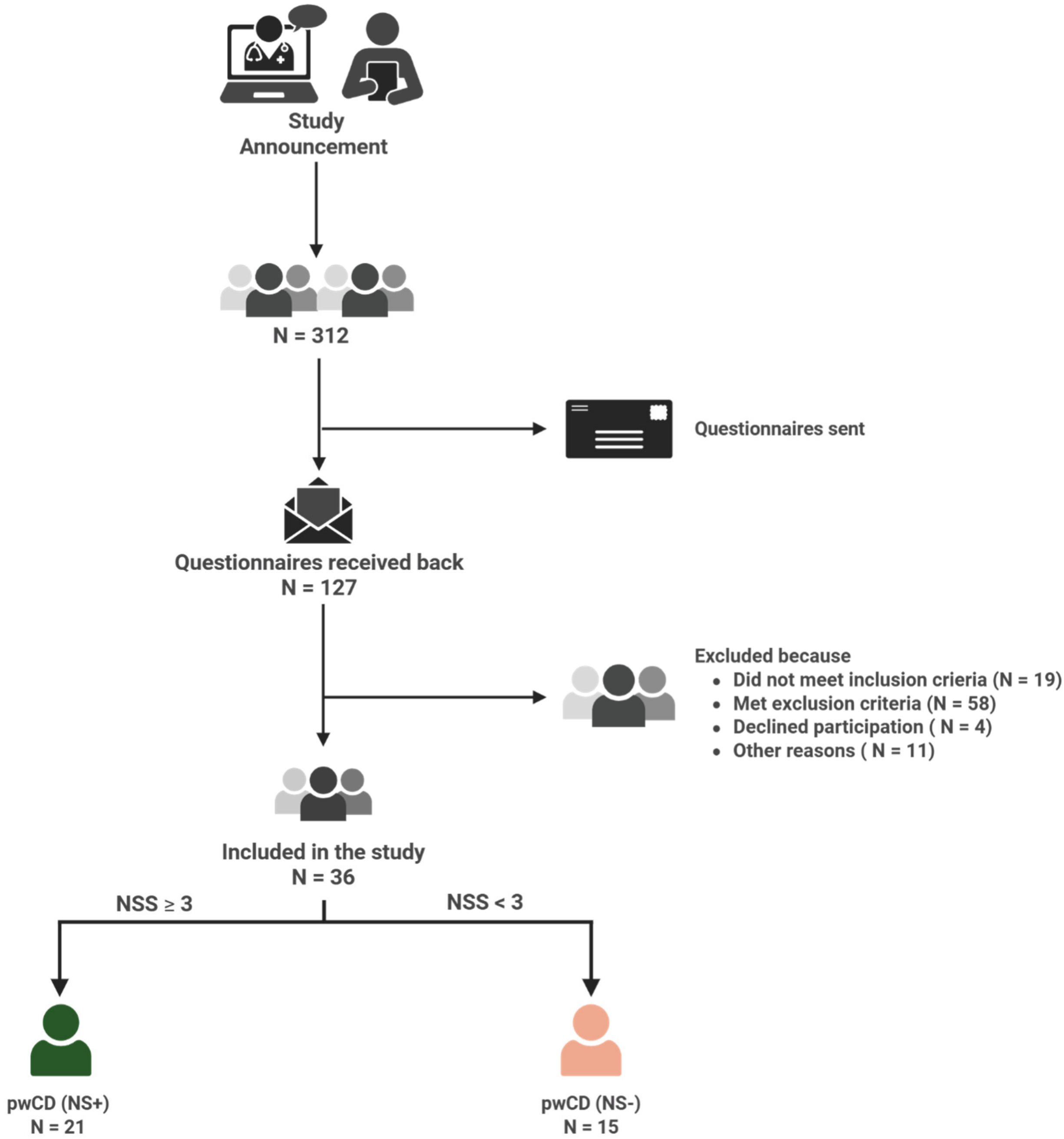

Suppl. Figure 2

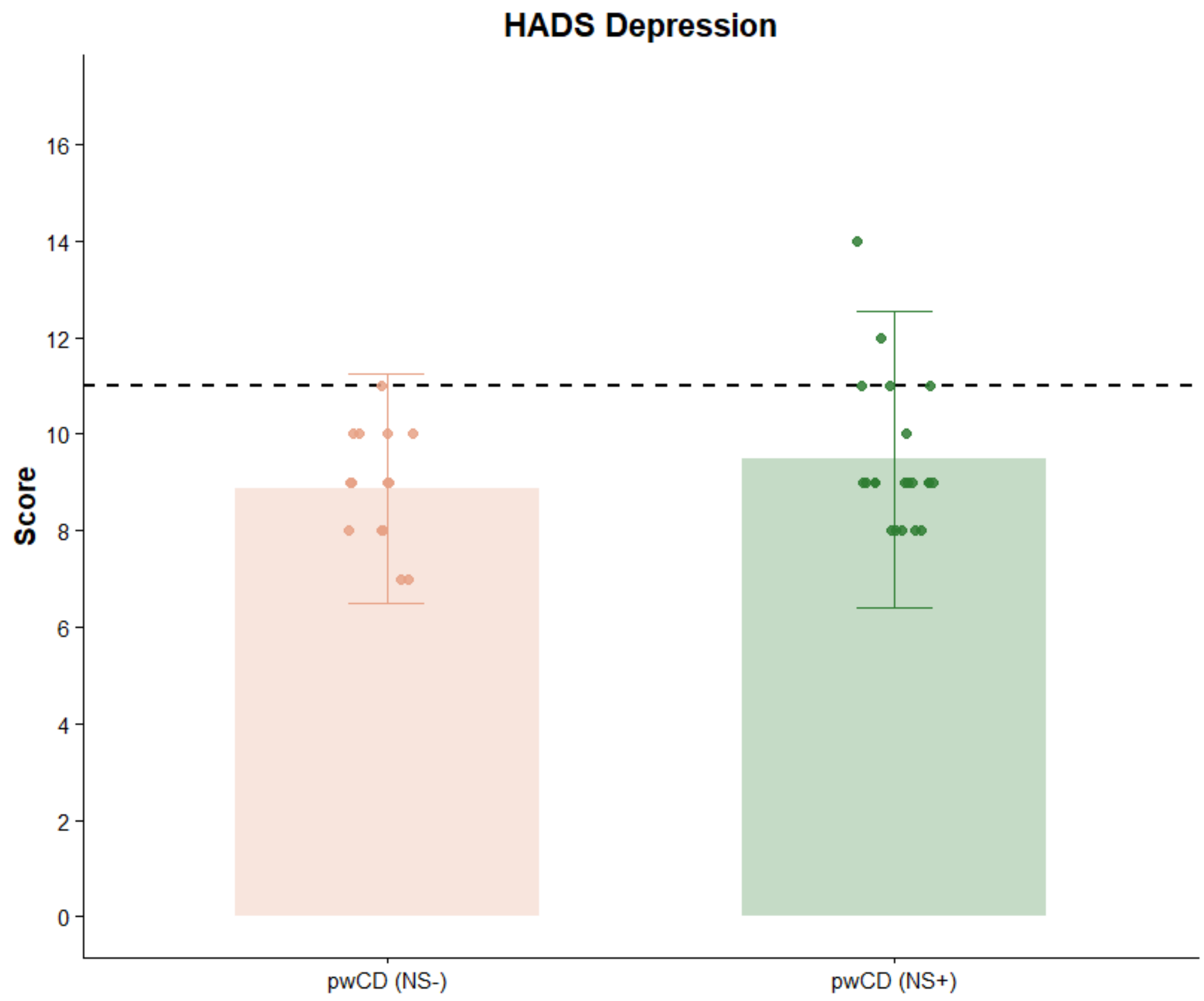

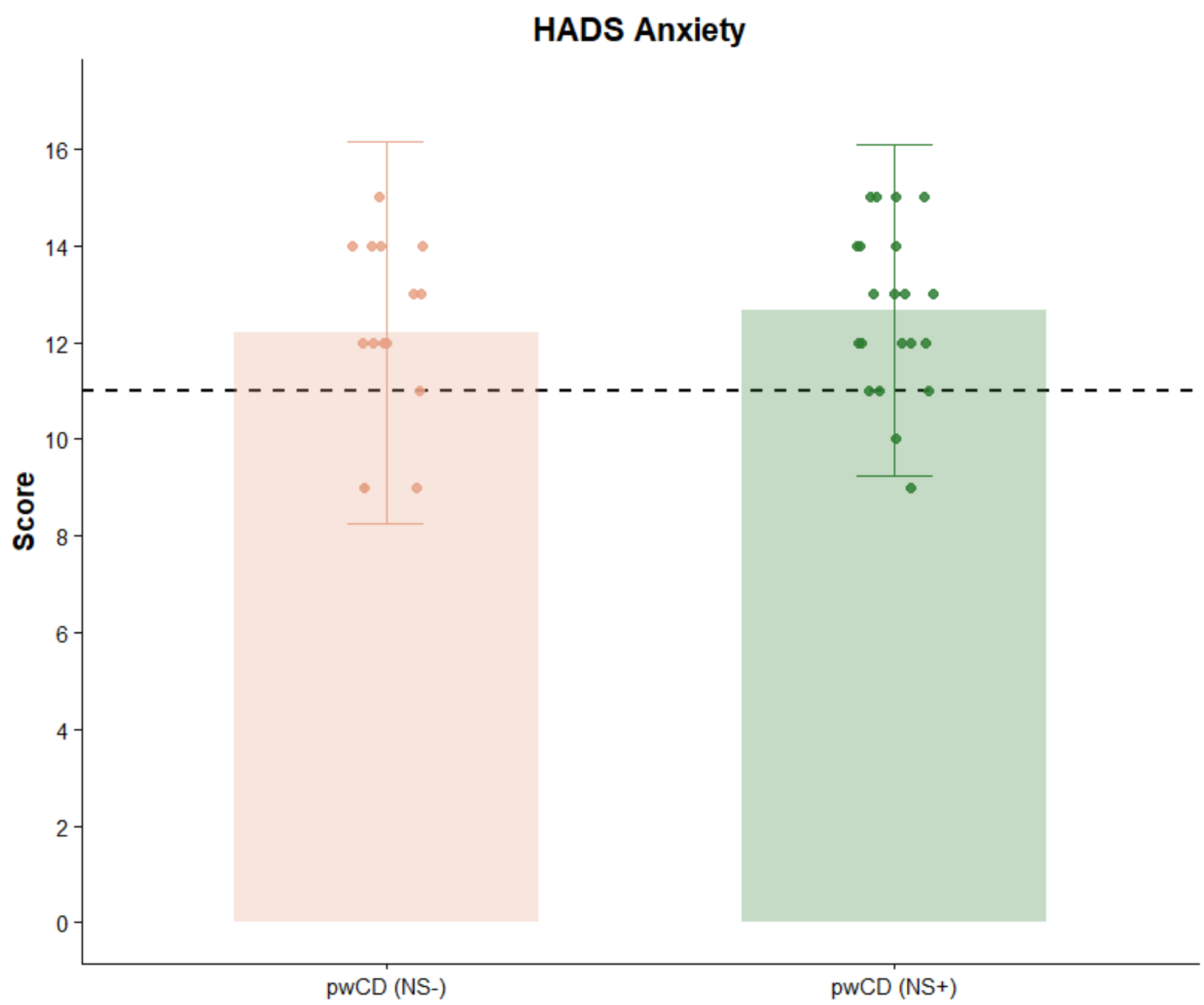

Suppl. Table 1

Variable	pwCD (NS+) (N=21)	pwCD (NS-) (N=15)
Reduced biceps reflexes	0.0% (N=0)	0.0% (N=0)
Reduced triceps reflexes	0.0% (N=0)	0.0% (N=0)
Reduced brachioradialis reflexes	0.0% (N=0)	0.0% (N=0)
Reduced patellar reflexes	4.8% (N=1)	0.0% (N=0)
Elbow flexor paresis	0.0 % (N=0)	0.0% (N=0)
Elbow extensor paresis	0.0 % (N=0)	0.0% (N=0)
Wrist extensor paresis	0.0 % (N=0)	0.0% (N=0)
Wrist flexor paresis	0.0 % (N=0)	0.0% (N=0)
Finger abductor paresis	4.8 % (N=1)	0.0% (N=0)
Finger flexor paresis	0.0 % (N=0)	0.0% (N=0)
Finger extensor paresis	0.0 % (N=0)	0.0% (N=0)
Thumb abduction paresis	9.5% (N=2)	0.0% (N=0)
Thumb adduction paresis	4.8 % (N=1)	0.0% (N=0)
Thumb opposition paresis	4.8 % (N=1)	0.0% (N=0)
Knee flexor paresis	0.0 % (N=0)	0.0% (N=0)
Knee extensor paresis	0.0 % (N=0)	0.0% (N=0)
Foot dorsiflexor paresis	0.0 % (N=0)	0.0% (N=0)
Foot plantar flexor paresis	0.0 % (N=0)	0.0% (N=0)
Toe extensor paresis	0.0 % (N=0)	0.0% (N=0)
Toe flexor paresis	0.0 % (N=0)	0.0% (N=0)

Suppl. Table 2

Variable	CD Patients (NS+) (N=21)	CD Patients (NS-) (N=15)
Hypaesthesia (Hand)	23.8% (N=5)	6.7% (N=1)
Hypaesthesia (Feet)	28.6% (N=6)	0% (N=0)
Pinprick Hyperalgesia (Hand)	14.3% (N=3)	0% (N=0)
Pinprick Hyperalgesia (Feet)	9.5% (N=2)	0% (N=0)
Pinprick Hypalgesia (Hand)	4.7% (N=1)	6.7% (N=1)
Pinprick Hypalgesia (Feet)	19% (N=4)	0% (N=0)
Dynamic Mechanical Allodynia	4.8% (N=1)	0% (N=0)
Pathological CDT (Hand)	23.8% (N=5)	26.6% (N=4)
Pathological WDT (Hand)	33.3% (N=7)	40% (N=6)
Pathological CDT or WDT (Hand)	47.6% (N=10)	46.7% (N=7)
Pathological CDT (Feet)	28.6% (N=6)	13.3% (N=2)
Pathological WDT (Feet)	28.6% (N=6)	13.3% (N=2)
Pathological CDT or WDT (Feet)	42.8% (N=9)	20% (N=3)
Pathological IENFD	92.8% (N=13/14)	40% (N=6/15)
Pathological AFT	0% (N=0)	0% (N=0)
